# Biosynthesis of unique natural product scaffolds by Fe(II)/αKG-dependent oxygenases

**DOI:** 10.1007/s11418-025-01880-z

**Published:** 2025-02-06

**Authors:** Takayoshi Awakawa

**Affiliations:** RIKEN Center for Sustainable Resource Science, Wako, Saitama 351-0198 Japan

**Keywords:** Biosynthesis, Oxidation, Fe(II)/αKG-dependent oxygenase, Meroterpenoid, Nonproteinogenic amino acid, Aziridine

## Abstract

Fe(II)/αKG-dependent oxygenases are multifunctional oxidases responsible for the formation of unique natural product skeletons. Studies of these enzymes are important because the knowledge of their catalytic functions, enzyme structures, and reaction mechanisms can be used to create non-natural enzymes through mutation and synthesize non-natural compounds. In this review, I will introduce the research we have conducted on two fungal Fe(II)/αKG-dependent oxygenases, TlxI-J and TqaL. TlxI-J is the first Fe(II)/αKG-dependent oxygenase type enzyme heterodimer that catalyzes consecutive oxidation reactions, hydroxylation followed by retro-aldol or ketal formation, to form the complex skeletons of meroterpenoids. TqaL is the first naturally occurring aziridine synthase, and I will discuss the mechanism of its unique C–N bond formation in nonproteinogenic amino acid biosynthesis. This review will advance research on the discovery of new enzymes and the analysis of their functions by reviewing the structures and functions of these extraordinary Fe(II)/αKG-dependent oxygenases, and promote their use in the synthesis of new natural medicines.

**Graphical Abstract:**

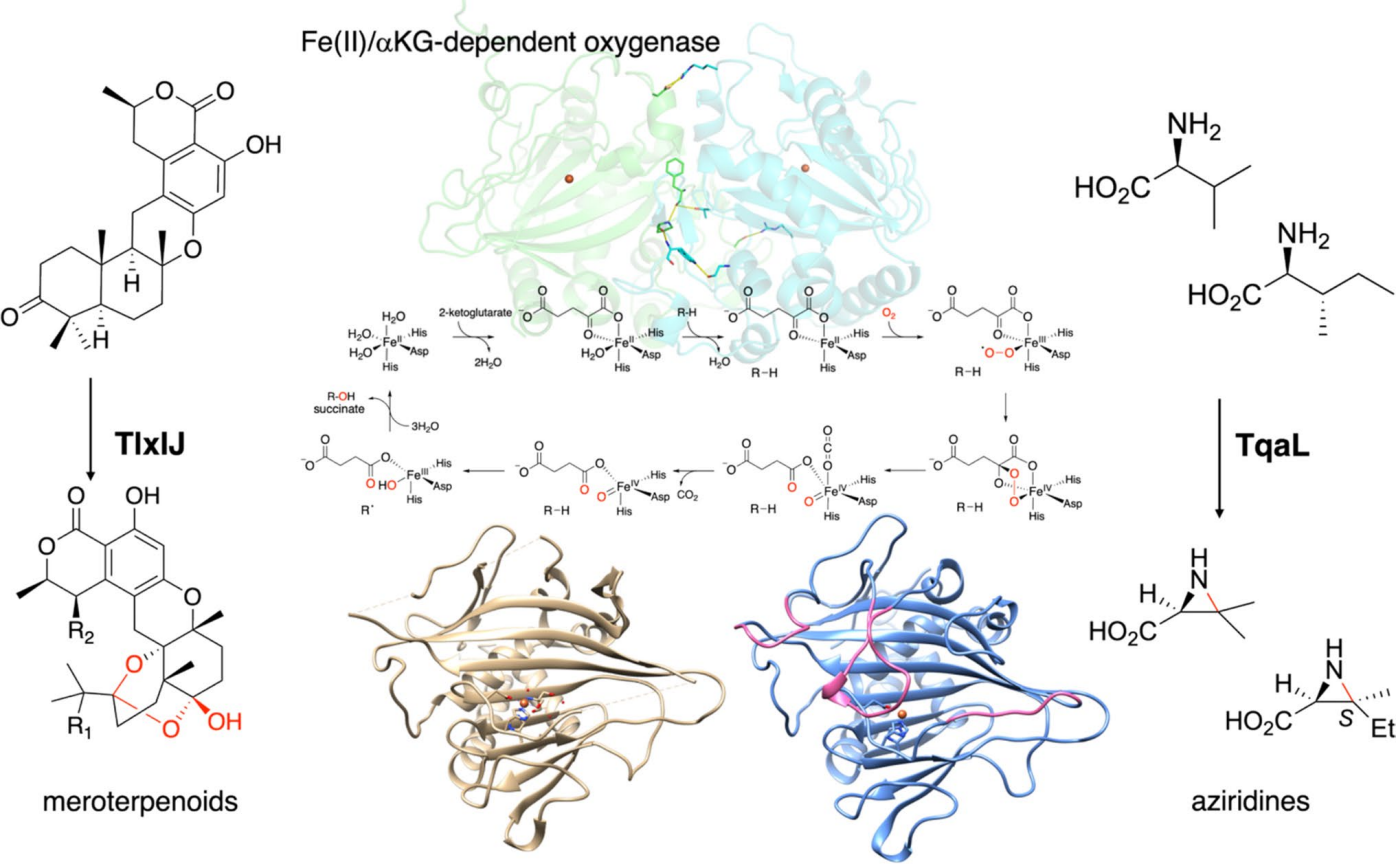

## Introduction

**Fig. 1:**
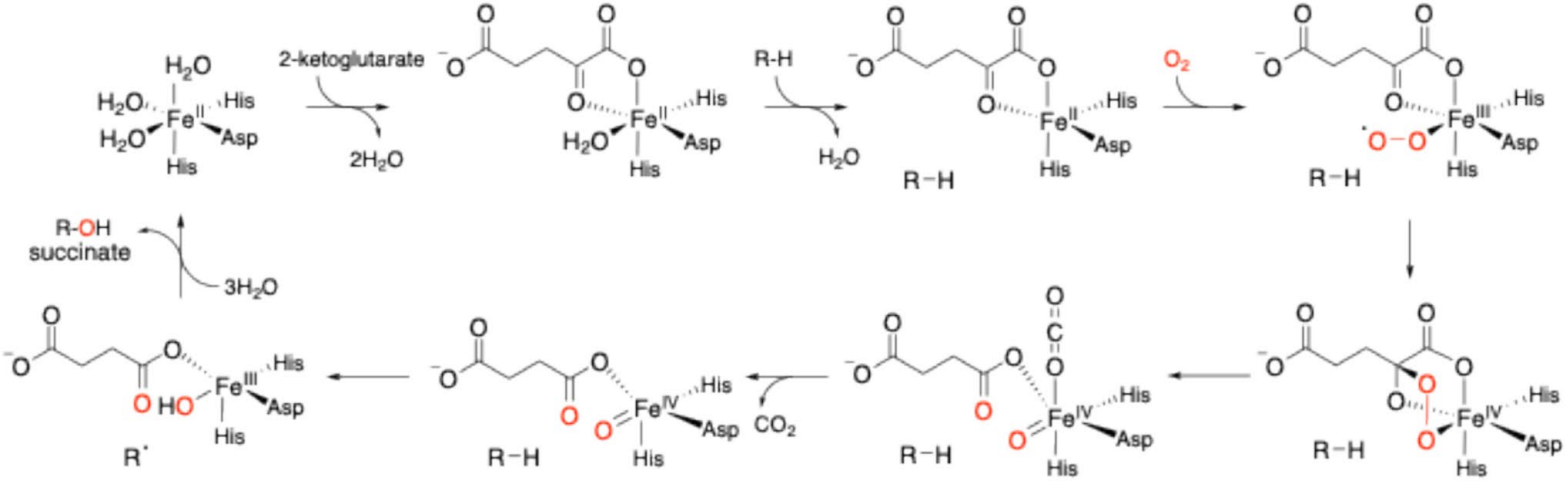
The catalytic cycle of Fe(II)/αKG-dependent oxygenases

Natural products remain important sources of pharmaceuticals [1]. However, the number of new antibiotics isolated from natural sources has decreased [2], and new methodologies for synthesizing natural products have become necessary. Although pharmaceutical production through organic synthesis features high reaction efficiencies and yields, production using biosynthetic enzymes is attracting keen attention as an alternative method. Biosynthetic enzyme reactions are environmentally friendly because they do not use organic solvents, and have the advantage of excellent regio- and stereospecificities. Some biosynthetic enzymes catalyze reactions that are also interesting from a chemical perspective, and investigations of these reactions and their detailed mechanisms are important not only from the viewpoint of medicinal chemistry, but also from the perspectives of enzyme chemistry and engineering. We have been studying oxidases and working to exploit biosynthetic enzymes for meroterpenoids, compounds with terpenoid substructures, and alkaloids in filamentous fungi [3–5]. Among these oxidases, we have focused on Fe(II)/α-ketoglutarate(αKG)-dependent oxygenases to identify novel enzyme reactions, analyze enzyme structures and functions, and elucidate catalytic mechanisms. The Fe(II)/αKG-dependent oxygenases catalyze the decarboxylation of αKG with Fe(II) to produce succinate and Fe(IV) = O, and the Fe(IV) = O then extracts a hydrogen atom from the substrate to produce a radical (Fig. 1) [6–10]. The generated radicals induce various chemical reactions with the substrate’s skeleton, including hydroxylation, desaturation, epoxidation, C-X(heteroatom) bond formation, and C–C bond reconstruction [6–12]. In this review, I describe our recent studies on the Fe(II)/ αKG-dependent oxygenases involved in fungal meroterpenoid and alkaloid synthesis.

## Unusual heterodimer of Fe(II)/ αKG-dependent oxygenase TlxI-J in the biosynthesis of fungal meroterpenoids talaromyolides

**Fig. 2:**
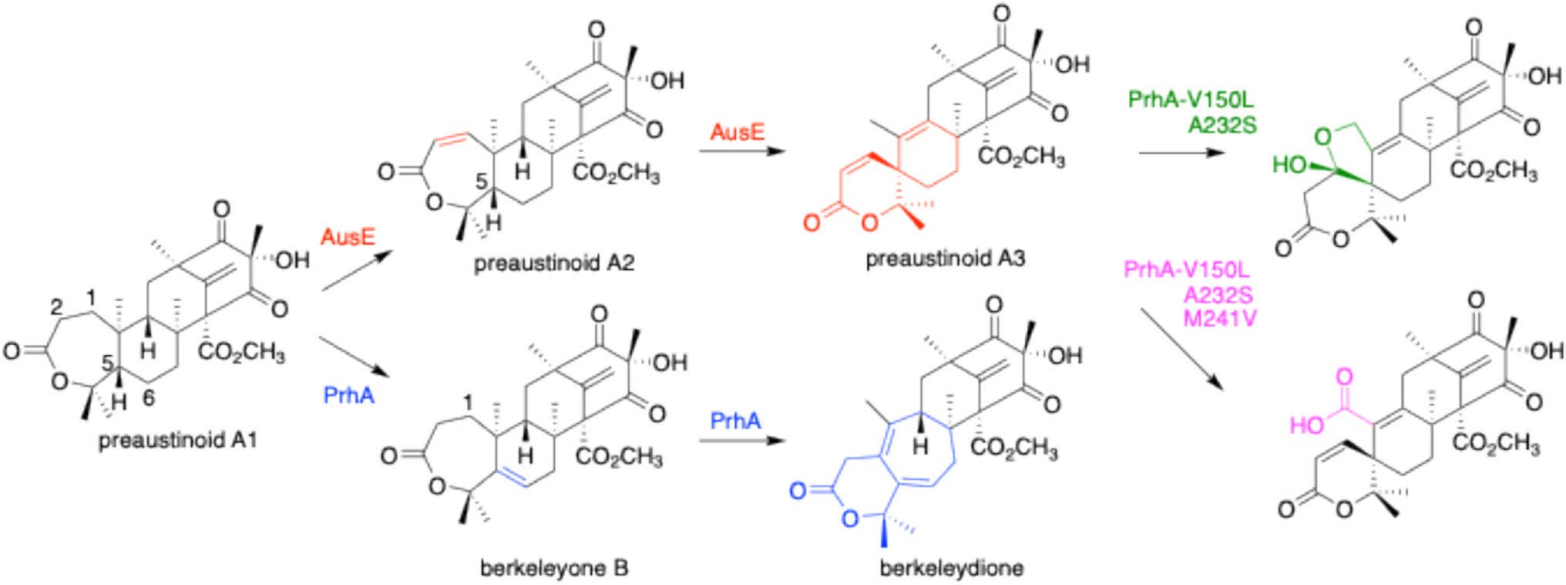
The reactions catalyzed by AusE, PrhA, and PrhA mutants from preaustinoid A1

We have discovered oxidases that catalyze unique reactions in the process of fungal meroterpenoid biosynthesis. Because the terpenoid portion of the substrate is highly flexible, radical formation in this portion gives rise to surprisingly complex skeletons [4, 5, 9]. For example, in the biosynthesis of the meroterpenoids austinol and paraherquonin, AusE and PrhA accept the substrate preaustinoid A1 as a common substrate, and synthesize the spirolatone protoaustinoid A3 and the heptadiene berkeleydione, respectively (Fig. 2) [13, 14].

**Fig. 3:**
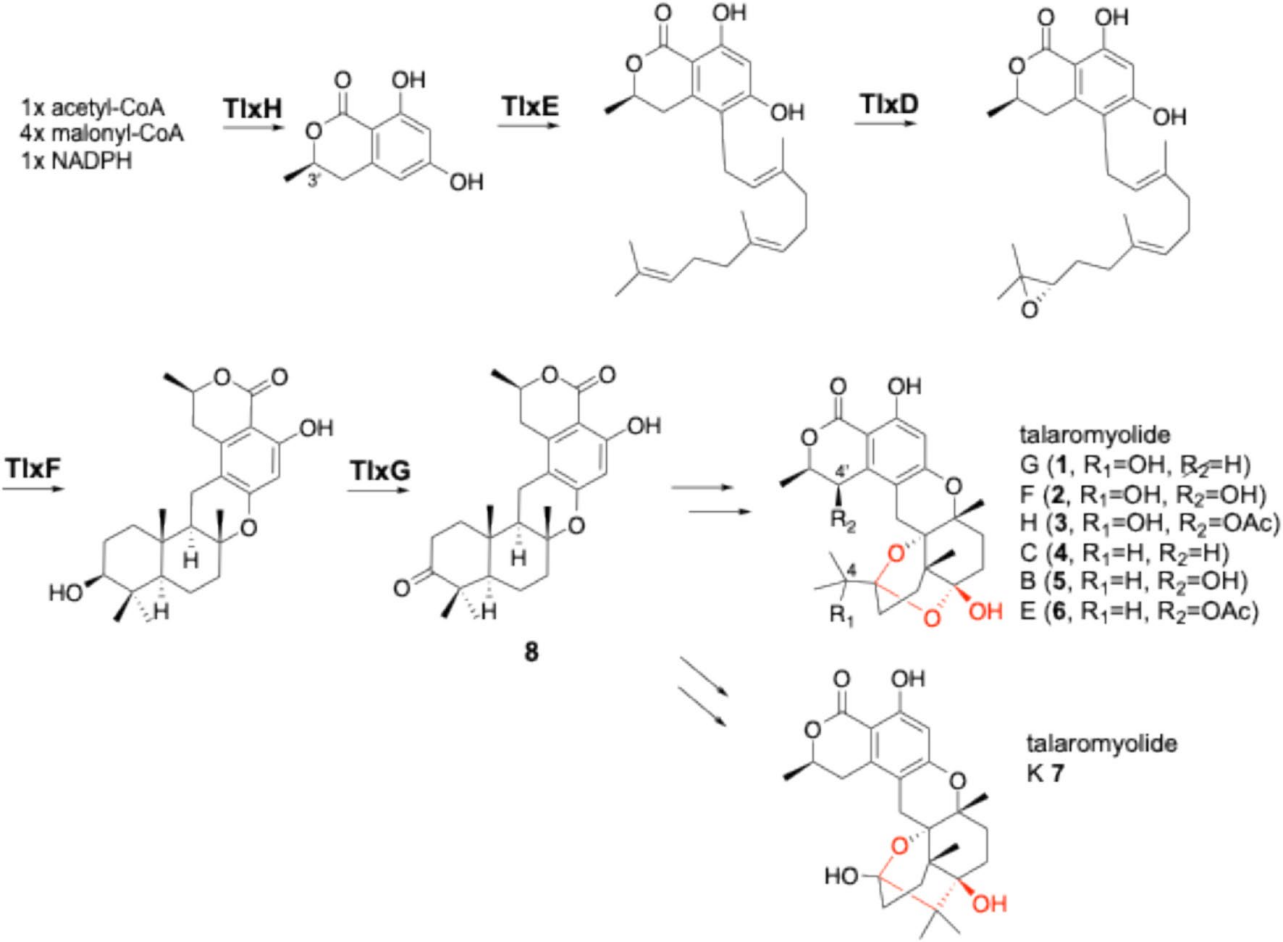
The early stage biosynthetic pathway of talaromyolides (**1**–**7**)

Although they share high homology (78%), slight differences in the amino acid residues at the substrate binding site affect the conformation of the substrate, resulting in different regioselective dehydrogenation reactions and distinct products. By solving the crystal structures of the enzymes and introducing targeted mutations into the active sites of AusE and PrhA, mutual conversion of products and even the synthesis of novel compounds were achieved (Fig. 2) [15]. Therefore, it is important to discover new enzymes and perform precise structural analyses in order to produce novel compounds. We have recently focused on the biosynthesis of talaromyolides **1**–**7**, which are 6/6/6/6/6/6 hexacyclic meroterpenoids with (3*R*)-6-hydroxylmellein and 4,5-seco-drimane structures isolated from marine algae-associated fungi [16, 17], and identified the Fe(II)/αKG-dependent oxygenases involved in the formation of their characteristic seco-drimane structures [18]. We firstly expressed the biosynthetic genes encoding the polyketide synthase (TlxH), UbiA-prenyltransferase (TlxE), flavin-dependent monooxygenase (TlxD), membrane-bound terpene cyclase (TlxF), and short-chain dehydratase/reductase (TlxG) in *Aspergillus oryzae* NSAR1 [19, 20] to produce intermediate **8** (Fig. 3).

**Fig. 4:**
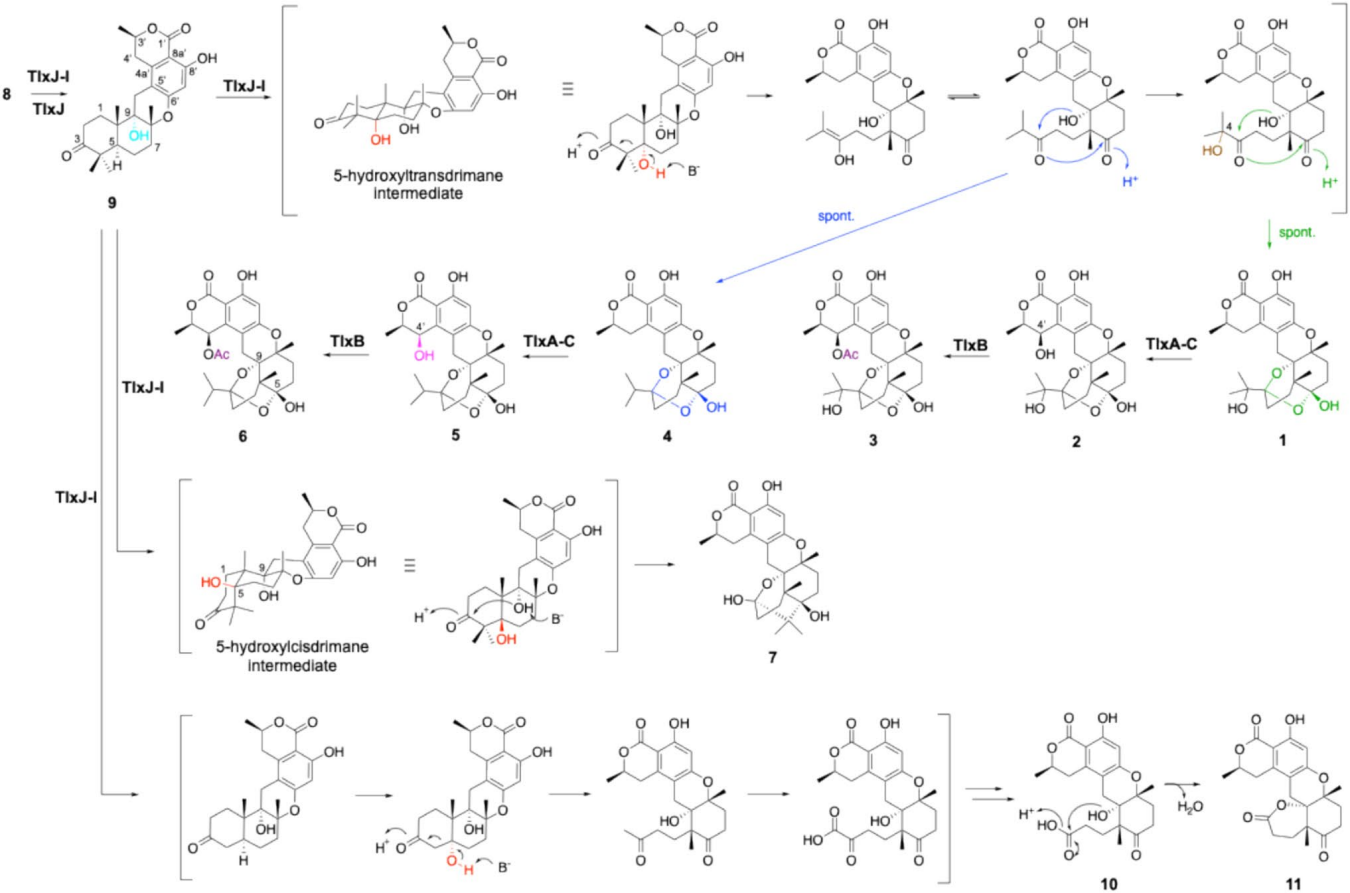
Reactions of monomer TlxJ and heterodimer TlxI-J to synthesize **1**–**7** and unnatural compounds **10** and **11**

The gene cluster includes four uncharacterized genes (TlxA/C/I/J) that encode Fe(II)/αKG-dependent oxygenases, and these are expected to be involved in the conversion of **8** to **1**–**7**. However, an in vitro assay using a His-tagged protein derived from *Escherichia coli* showed that TlxJ hydroxylated C-9 of **8** to yield **9** (Fig. 4), while no other enzymes catalyzed the subsequent oxidation reactions.

**Fig. 5:**
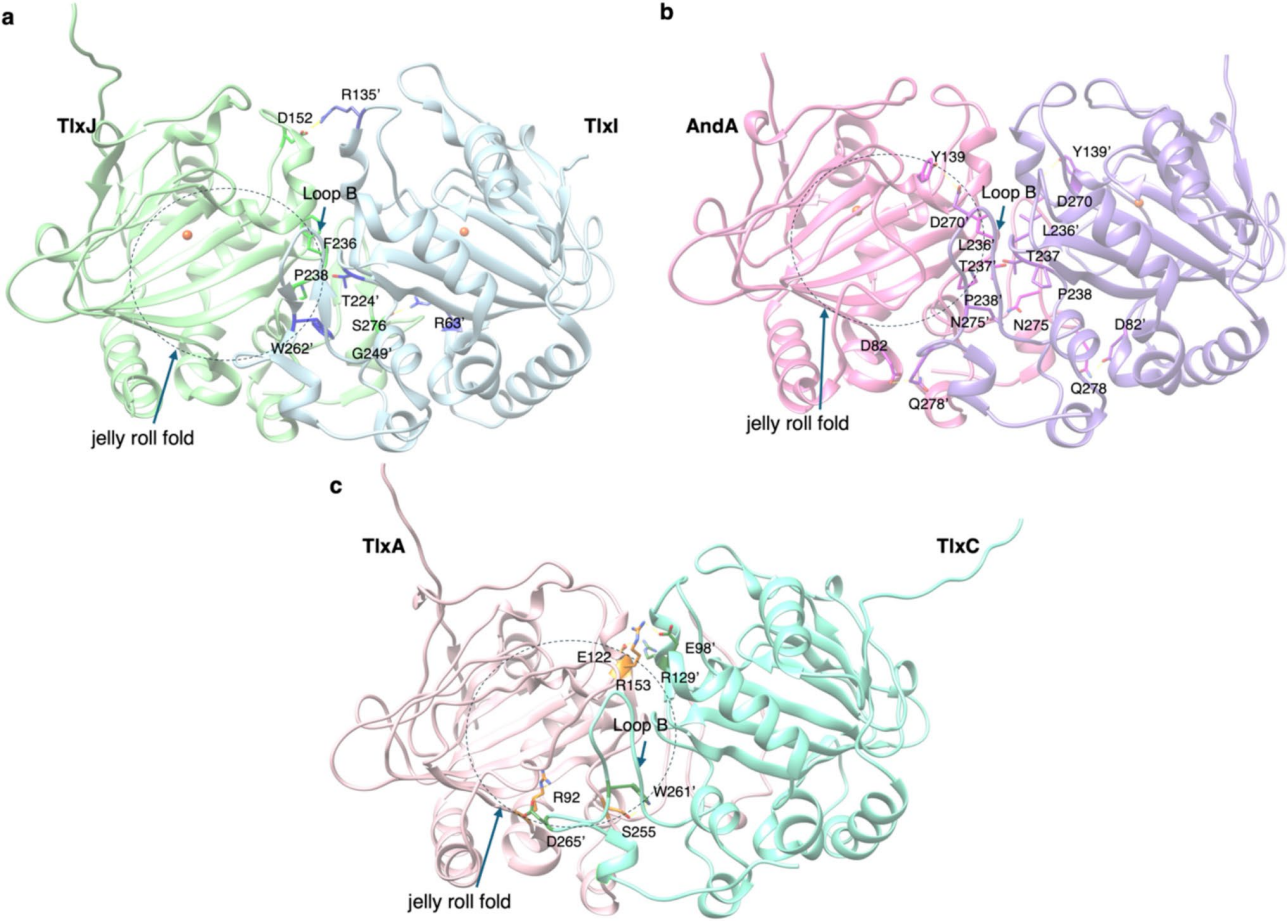
The comparison of the X-ray crystal structures of heterodimer TlxI-J (PDB: 7VBQ) (a), homodimer AndA (PDB: 5ZM3) (b), and the alpha fold model of heterodimer TlxA-C (c)

We then examined the amino acid sequences of TlxA/C/J/I in detail, and found that TlxI lacked the arginine residue involved in retaining αKG and LoopA, which serves as a lid for the substrate-binding pocket. Thus, we predicted that TlxI might act as a chaperone for TlxJ, which is expressed at low levels in *E. coli*. By co-expressing TlxJ and TlxI, the level of protein expression was greatly improved compared to that when TlxJ was expressed alone. Based on these data, we thought that TlxI might contribute to the structural stabilization of TlxJ. In fact, pull-down assays and gel filtration chromatography revealed that TlxJ and TlxI maintain a strong dimer structure. Remarkably, TlxI-J accepted **9** and further oxidized it to talaromyolides G (**1**), C (**4**), and K (**7**) (Fig. 4). In addition, this reaction generated the new products **10** and **11**. The results showed that TlxI-J hydroxylated C-5 of **9**, and the retro-aldol reaction led to the opening of the A ring of the dorimane ring, yielding **4**, which was then further hydroxylated at C-4, giving **1** (Fig. 4). Hydroxylation of C-5 will also produce **7**, but the difference is that **1** and **4** are produced from trans-5-hydroxydrimane, while **7** is produced from cis-5-hydroxydrimane. In **10** and **11**, the two methyl groups of **9** become carboxylic acids after six oxidations, and the decarboxylated intermediate undergoes C-5 hydroxylation, opening the A ring. The methyl groups of the resulting compound are converted to carboxylic acids after three more oxidations, producing **10** via decarboxylation, which is then esterified to produce **11** (Fig. 4). The ten oxidation reactions indicate that the enzyme accepts different intermediates multiple times and highlights the tolerance of TlxI-J’s substrate specificity. In addition, TlxA-C, in which TlxC serves a chaperone-like role similar to TlxI, also formed a heterodimer and catalyzed the hydroxylation of C-4’ to produce **2** and **5** from **1** and **4**, respectively. Gene deletions of TlxI and TlxC confirmed that they are essential for the biosynthesis of **1**–**7**, and are also essential for biosynthesis in vivo. The crystal structure of TlxI-J was solved and the alpha fold model of heterodimer TlxA-C was constructed, and its heterodimer structure was compared with the homodimeric structure of AndA, an Fe(II)/αKG-dependent oxygenase responsible for the biosynthesis of the meroterpenoid anditimin [21]. As a result, the modes of dimer formation for homodimers and heterodimers are different (Fig. 5). The three models are similar in that LoopB of one of the monomers forms a substrate binding pocket with a jelly roll fold, but in the homodimer, two pockets are formed per dimer, while in the heterodimer, only one is formed. The hydrogen bond between D82 and Q278 in AndA is in the same position as the hydrogen bond between R92 and D265’ in TlxA-C, helping to form the substrate binding pocket. The hydrogen bonds between D152 and R135’ in TlxI-J and between E122 and R129’ in TlxA-C are in the same position in the upper right of the jelly roll fold. But there is no such bond in AndA, and instead a hydrogen bond between Y139 and D270’ is present, strengthening the bond between loop B and the jelly roll fold.

TlxI-J and TlxA-C are the first reported Fe(II)/αKG-dependent oxygenase family enzymes that form heterodimer structures. In addition to the reports of heterodimer formation by the ketosynthase in the type II PKS system [22], the amino acid synthases HisFH [23] and TrpAB [24–27], and the hinokiresinol synthase HRSαβ [28], heterodimer formation by the non-heme iron (NHI) enzyme MbnB and auxiliary protein MbnC has also been reported recently. MbnBC is responsible for producing oxazole and thioamide in methanobactin biosynthesis [29]. MbnB requires MbnC to be stably expressed in *E. coli*, and the substrate peptide MbnA binds to the interface between MbnB and MbnC [30], suggesting the importance of dimer formation in catalysis. Homologs of MbnBC are involved in the biosynthesis of various ribosomal peptide compounds, including TgHI in thioglutamate biosynthesis [31] and ChrHI in chryseobasin biosynthesis [32], and it is expected that additional homologous proteins will be discovered in future research.

## Aziridine forming Fe(II)/ αKG-dependent oxygenases TqaL in the biosynthesis of fungal non-proteinogenic amino acids.

**Fig. 6:**
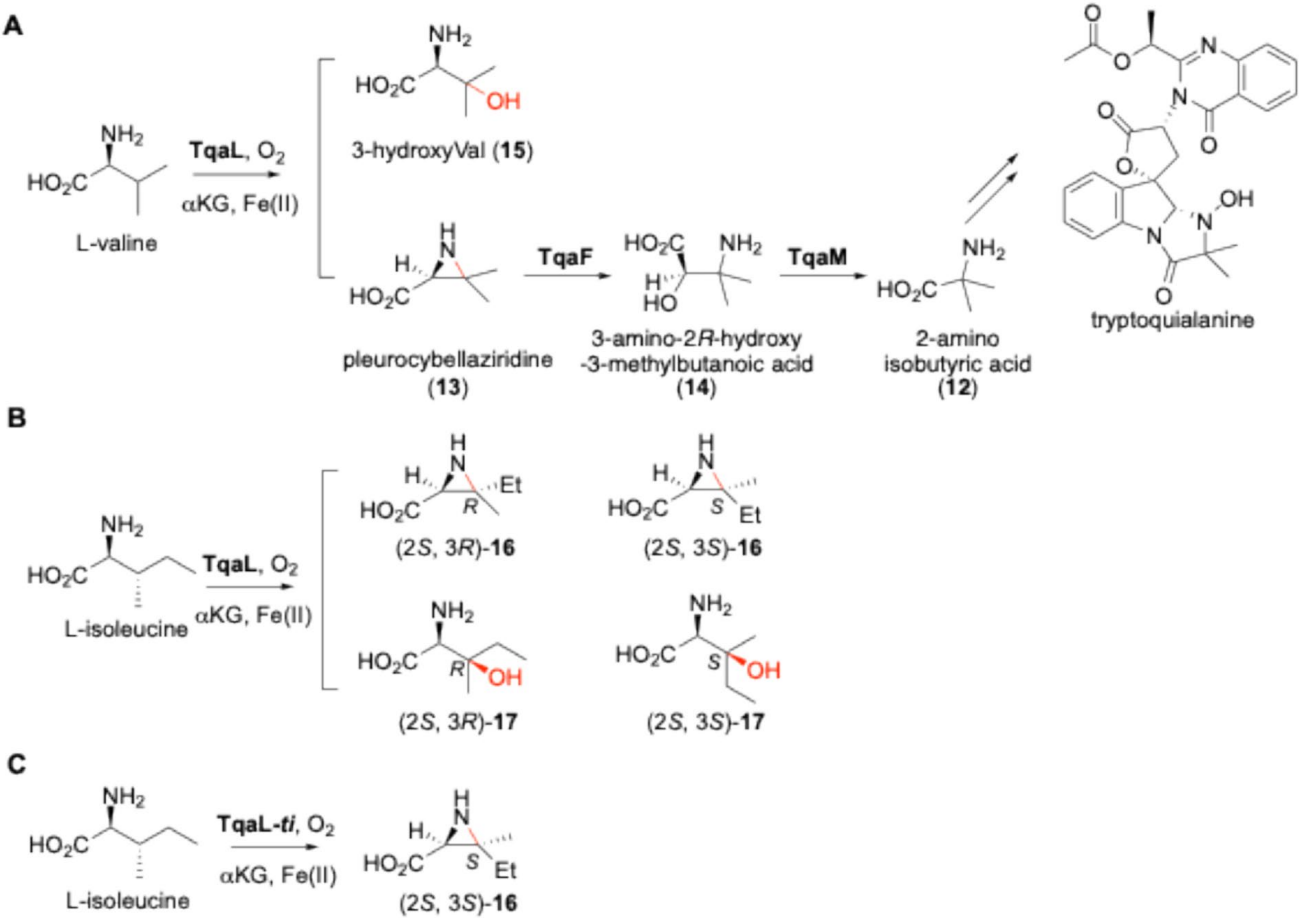
The oxidations of L-Val by TqaL (A), L-Ile by TqaL (B), and L-Ile by TqaL-*ti* (C)

In addition to meroterpenoids, Fe(II)/αKG-dependent oxygenases catalyze unique reactions in the biosynthesis of non-proteinogenic amino acids. For 2-aminoisobutyric acid (AIB, **12**) synthesis in alkaloid tryptoquialanine biosynthesis in *Penicillium*, the Fe(II)/αKG-dependent oxygenase TqaL and the NHI-dependent oxygenase TqaM were identified by gene deletion experiments [33], but their reactions have not been identified. We predicted that **12** is biosynthesized via pleurocybellaziridine (**13**) from L-valine, based on the report that **13** has been isolated from the basidiomycete fungus *Pleurocybella porrigens* [34]. We prepared TqaL and TqaM as His-tagged recombinant proteins from *E. coli*, performed the reaction using L-valine as a substrate, and determined the structure of the product from the TqaL reaction by comparison with the standard. The data revealed that TqaL accepts L-valine to yield the aziridine ring of **13** (Fig. 6A) [35].

This is the first report of an aziridine synthase in nature. In recent years, several aziridine synthesis reactions have been described, including competing intramolecular displacement reactions and reactions mediated by sulfotransfer in the azabicyclo[3.1.0]hexane (ABCH) ring in ficelomycin biosynthesis [36–38], but this was the first report of an aziridine synthase. Our detailed bioinformatics analysis demonstrated that the haloalkanic acid dehalogenase TqaF, in the Tqa cluster, is likely to be an aziridine hydrolase. As expected, TqaF catalyzed the attack of water at C-2 of 13 to open the ring in an SN2 manner and produced 3-amino-2*R*-hydroxy-3-methylbutanoic acid (**14**) in vitro. TqaM accepted **14** and catalyzed decarboxylative oxidation to produce **12** (Fig. 6A).

**Fig. 7:**
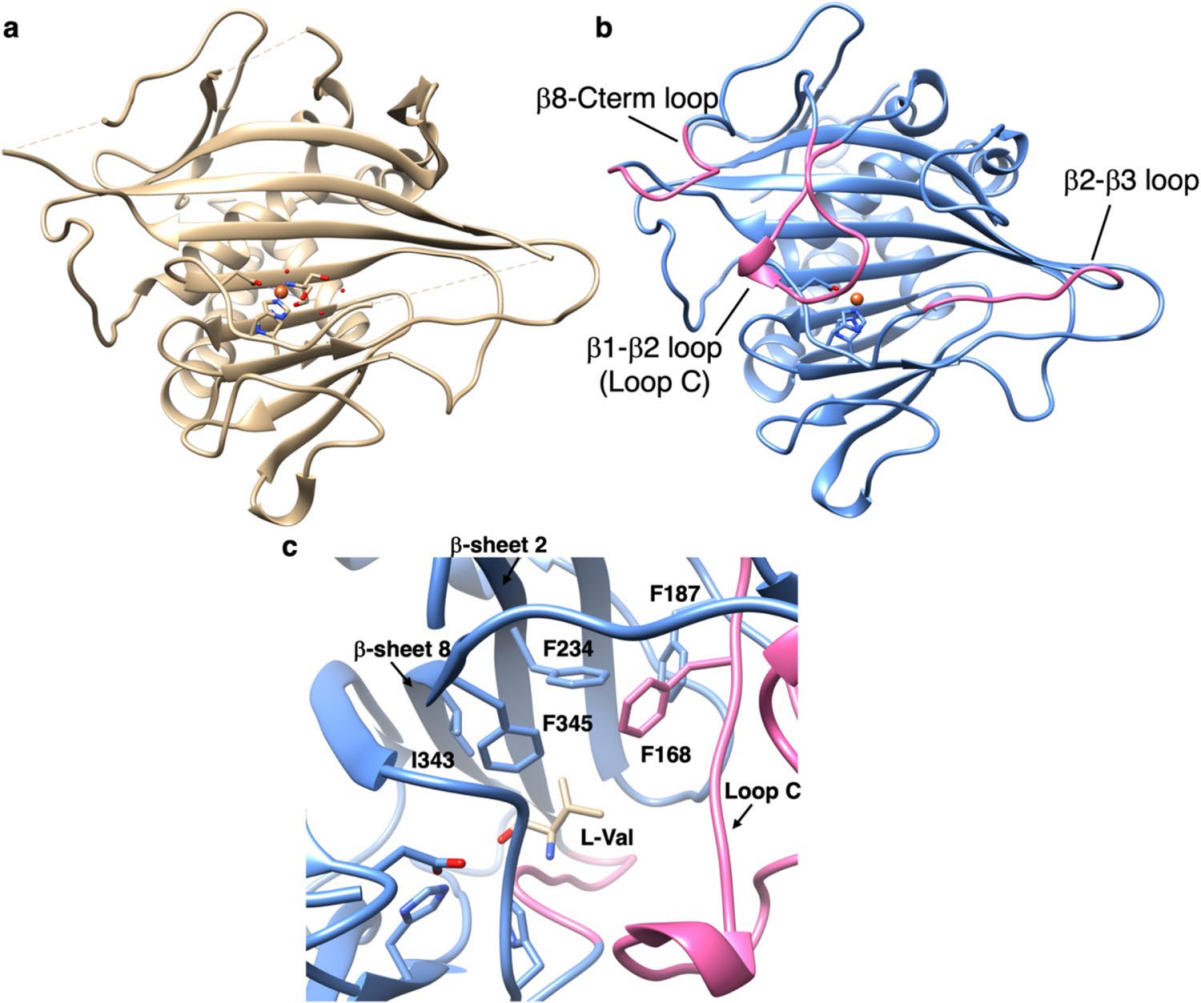
Protein structures of TqaL. X-ray crystal structure of apo-TqaL (PDB: 7EEH) (a), the alpha fold model of TqaL with Fe ion (b), the close up view of the alpha fold model of TqaL docked by L-Val (c)

The X-ray crystal structure of apo-TqaL revealed that it adopts a jelly-roll fold, as seen in other Fe(II)/αKG-dependent oxygenases, but the structure of loops for substrate binding was missing (Fig. 7A). Thus, we used AlphaFold2 to construct a structural model of the enzyme, and identified the amino acid residues responsible for substrate binding by docking L-valine into the cavity (Fig. 7B) [39].

The model suggested that hydrophobic residues, including F168 and F187 on loopC, F234 on β-sheet 2, and I343 and F345 on β-sheet 8, are located close to the side chain of L-valine, suggesting their importance for substrate binding (Fig. 7c). The F168L, F187L, and F234L mutations led to a loss of activity, while I343A produced a more efficient enzyme; however, the mutant enzyme yielded more 3-hydroxyvaline (**15**) than WT. Remarkably, F345L and F345A generated **15** as the major product instead of **13**. These data suggest the importance of these residues in controlling substrate binding and reactivity. Among them, F345 was the crucial residue that governs aziridine formation over hydroxylation. The substrate specificity of TqaL was investigated by applying 20 proteinogenic α-amino acids as substrates, and L-Ile was identified as the only accepted substrate besides L-Val. The TqaL reaction with L-Ile yielded two stereoisomers of aziridines, (2*S*, 3*S*)-**16** and (2*S*, 3*R*)-**16**, and two stereoisomers of 3-hydroxylated isoleucine, (2*S*,3*S*)-**17** and (2*S*, 3*R*)-**17** (Fig. 6B). These data suggested that, of the two substrates, L-Val is strictly recognized and its reaction is controlled, while the substrate recognition of L-Ile is less stringent. Conversely, the recently discovered TqaL from *Tolypocladium* (TqaL-*ti*) showed strict recognition for L-Ile and produed only (2*S*, 3*S*)-**16**, indicating the different substrate recognition modes for these two TqaL enzymes (Fig. 6C) [40].

**Fig. 8:**
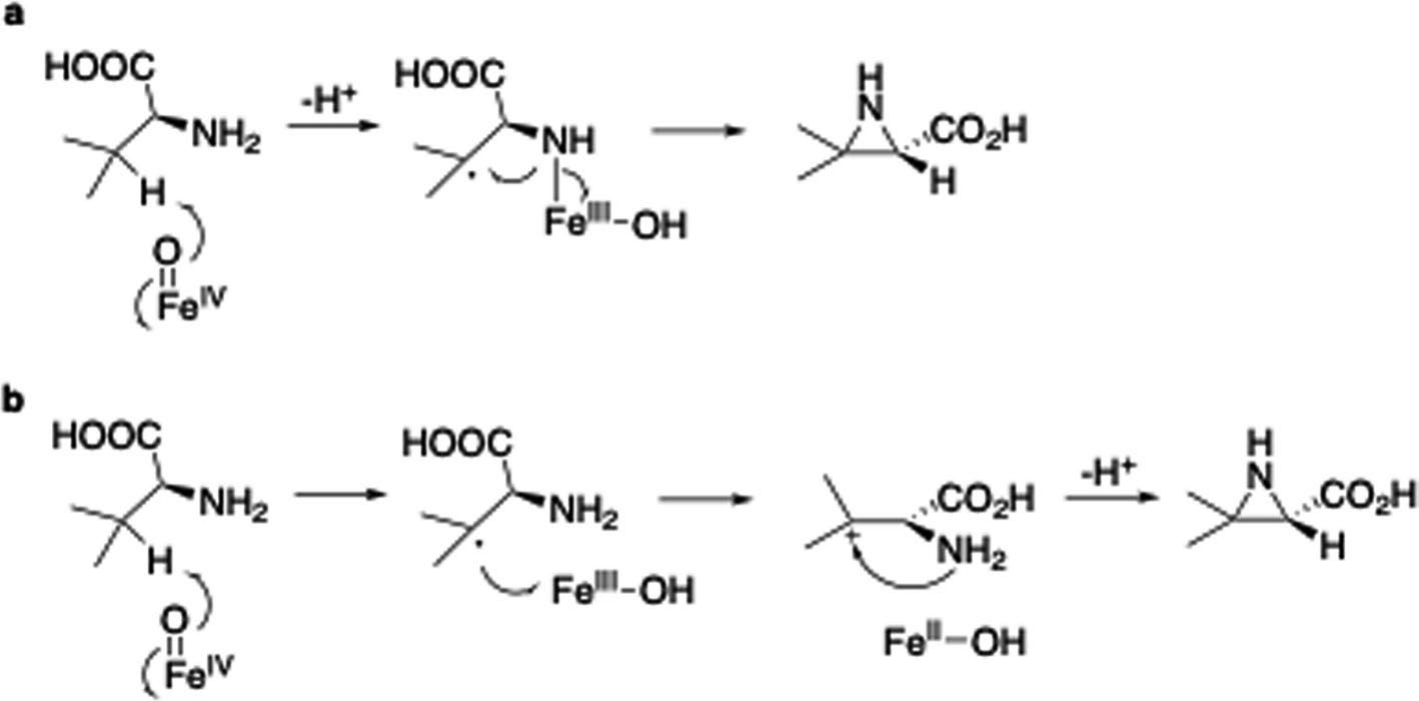
The proposed reaction mechanism of aziridine synthesis by TqaL. A radical mechanism is depicted in a, and a polar mechanism is depicted in b

TqaL is the only NHI enzyme reported thus far that catalyzes the formation of aziridines, although several NHI enzymes catalyze oxidative ring formation, such as cyclization and cyclopropanation [8, 41, 42]. Therefore, the reaction mechanism of TqaL is interesting. At first, it was thought that the radical at C-3 was generated by Fe(IV) = O, but two possibilities were then considered: the formation of a cation via the transfer of the radical to Fe(III) (polar mechanism), or the reaction of the diradical via the abstraction of a hydrogen atom from the amino group (radical mechanism) (Fig. 8).

Dr. Wei-chen Chang’s group conducted TqaL assays using several valine analogs, which revealed that TqaL accepts homoalanine as a substrate and catalyzes its hydroxylation [43]. Based on these data, isotope tracing experiments using H_2_^18^O and ^18^O_2_, and quantitative product analysis, they proposed that the polar mechanism is more likely. Dr. Winju Wang’s group also evaluated this reaction mechanism by an MD simulation and quantum mechanical/molecular mechanical (QM/MM) analyses [44]. Their computational studies supported the radical mechanism, and the calculated structure of the Fe-Val radical intermediate suggests that the reaction of the radical with the amine nitrogen is reasonable. Their simulation suggested that the radical pathway is more likely.

## Conclusion

This review has discussed the structures and functions of the unique Fe(II)/αKG-dependent oxygenases TlxI-J and TqaL, and introduced important findings for understanding their catalytic reactions. TlxI-J catalyzes C-5 hydroxylation to produce trans-5-hydroxydrimane, and **1**–**6** are generated by the subsequent retroaldol reaction, but at the same time, cis-5-hydroxydrimane is synthesized, and **7** is produced by the subsequent ketal formation. The differentiation between trans-5-hydroxydrimane and cis-5-hydroxydrimane by TlxI-J is intriguing. Furthermore, TlxI-J catalyzes ten oxidations of **8** to synthesize the unnatural compounds, **10** and **11**. The reported formation of TlxI-J heterodimers is the first for a Fe(II)/αKG-dependent oxygenase-type enzyme, and its evolution is interesting. Recently, the fusion enzyme TalA, which corresponds to TlxI-J, was reported [45], suggesting the co-evolution of TlxJ and TlxI. Looking at other examples of heterodimers, besides HRSαβ from plants, related enzyme genes are often discovered in microbial gene clusters. For species other than microorganisms, new heterodimers may be found by tagging one of the proteins and performing a pull-down assay, or focusing on homologous proteins with no function based on genomic information. The substrate binding site of TlxI-J is located in the region between the TlxI-J interactions, and adjusting the interaction forces may be effective for functional modification. Several Fe(II)/αKG-dependent oxygenases involved in amino acid oxidation have recently been discovered, and are being used to produce alkaloid or peptide compounds [11]. TqaL is the first identified enzyme that synthesizes an aziridine skeleton from amino acids, and various homologs have been discovered. Since many TqaL homologs exist in the filamentous fungus genome database, it is highly likely that TqaLs with different substrate specificities and reactivities will be discovered in the future. These sequence comparisons will help us to better understand why aziridine synthesis is catalyzed, rather than hydroxylation by hydroxyl rebound, which seems to proceed more quickly. To determine this mechanism, it will be necessary to conduct experiments using synthetic chemistry to analyze reactions using isotope-labeled substrate analogs, as well as computational science experiments to build and analyze the enzyme and intermediate complexes that undergo successive reactions. In synthetic chemistry, aziridines can be used in addition reactions to unusual quaternary carbons due to their ring strain, so their stereoselective synthesis by enzymes is likely to generate keen interest [46]. Several aziridine compounds have been isolated in nature, and it is highly likely that novel aziridine synthases will be found in their producers [47, 48]. In this review, I have outlined the structures, reactions, and catalytic mechanisms of Fe(II)/αKG-dependent oxygenases that synthesize interesting skeletons. This information will promote the synthesis of new peptides and alkaloids using biosynthetic enzymes, and further advance the discovery of natural medicines.
